# Universal slip dynamics in metallic glasses and granular matter – linking frictional weakening with inertial effects

**DOI:** 10.1038/srep43376

**Published:** 2017-03-06

**Authors:** Dmitry V. Denisov, Kinga A. Lőrincz, Wendelin J. Wright, Todd C. Hufnagel, Aya Nawano, Xiaojun Gu, Jonathan T. Uhl, Karin A. Dahmen, Peter Schall

**Affiliations:** 1 Institute of Physics, University of Amsterdam, P.O. Box 94485, 1090 GL Amsterdam, The Netherlands; 2 Department of Mechanical Engineering, Bucknell University, One Dent Drive, Lewisburg, PA 17837; 3 Department of Chemical Engineering, Bucknell University, One Dent Drive, Lewisburg, PA 17837; 4 Department of Materials Science and Engineering, Johns Hopkins University, Baltimore, MD 21218; 5 Department of Mechanical Engineering, Johns Hopkins University, Baltimore, MD 21218; 6 Department of Physics, University of Illinois at Urbana Champaign, 1110 West Green Street, Urbana, IL 61801; 7 Retired, Los Angeles, CA, USA.

## Abstract

Slowly strained solids deform via intermittent slips that exhibit a material-independent critical size distribution. Here, by comparing two disparate systems - granular materials and bulk metallic glasses - we show evidence that not only the *statistics* of slips but also their *dynamics* are remarkably similar, i.e. independent of the microscopic details of the material. By resolving and comparing the full time evolution of avalanches in bulk metallic glasses and granular materials, we uncover a regime of universal deformation dynamics. We experimentally verify the predicted universal scaling functions for the dynamics of individual avalanches in both systems, and show that both the slip statistics and dynamics are independent of the scale and details of the material structure and interactions, thus settling a long-standing debate as to whether or not the claim of universality includes only the slip statistics or also the slip dynamics. The results imply that the frictional weakening in granular materials and the interplay of damping, weakening and inertial effects in bulk metallic glasses have strikingly similar effects on the slip dynamics. These results are important for transferring experimental results across scales and material structures in a single theory of deformation dynamics.

The question of universality represents a grand challenge in materials deformation, which has been traditionally described by material-specific relations and mechanisms. The existence of universal scaling relations, if confirmed experimentally, would provide a novel means to connect microscopic degrees of freedom to macroscopic stress-strain response in a single theory of deformation across a wide range of solid materials. Recently, power-law statistics discovered in the stress fluctuations of slowly deformed single crystals^1^,^2^, bulk metallic glasses (BMGs)^3^,^4^, rocks^5^,^6^, granular materials^7^,^8^,^9^,^10^,^11^,^12^ and even earthquakes^13^,^14^,^15^,^16^ reveal similar strongly correlated deformation, suggesting underlying universal scaling relations in the slow deformation of disordered solids. These distributions are also well described by a mean-field model of elasto-plastic deformation^17^, in which the material’s elasticity causes coupling between locally yielding regions resulting in slip avalanches with intermittency as observed in the experiments. A recent comparison of widely different systems showed that indeed the magnitude of slip sizes exhibits very similar power-law statistics across a wide range of length scales from nanometers to kilometers^6^, as adequately described by the mean-field model, lending credence to the idea of underlying universal slip statistics. Yet, simple models suggest that while the slip *statistics* may be universal, the slip *dynamics*, i.e. the time evolution of slips might be material dependent and fall into different universality classes due to the different material-dependent deformation mechanism. Most notably the role of inertia and overdamping in classifying the microscopic dynamics remains unclear. These properties are particularly disparate in bulk metallic glasses and granular materials, where the constituent units are atoms and macroscopic particles, respectively, though a possible analogy in their deformation mechanism due to the randomly packed structure of both systems was pointed out^18^. Until now, the slip dynamics have been prohibitively difficult to compare between materials as the required time resolution is challenging to reach. Establishing the similarity of not only the statistics, but also the dynamics of slip avalanches would clarify the microscopic slip mechanism, and could greatly extend the claim of universality.

In this paper we provide the first evidence of strong similarity between slip dynamics in bulk metallic glasses^19^ and granular materials^7^, in which high-resolution stress measurements are possible. We show that despite the large differences in the nature of the two materials in terms of the size, interactions, and dynamics of the constituent particles, they share a regime with identical rescaled stress fluctuations, temporal profiles, and dynamics, in agreement with the predictions of mean-field theory. The observed agreement in the slip dynamics of bulk metallic glasses and granular materials shows that universality may extend beyond the slip statistics alone to also include the dynamics. The results are consistent with the predictions of a simple coarse grained model for slip avalanches originating from weak spots in the material. Besides the universal regime, we also delineate a non-universal regime of large system-spanning slips, with system-specific statistics, governed by boundary conditions and finite-size effects. This first rigorous comparison of not only slip statistics but also slip dynamics in two fundamentally disparate systems suggests a universal scaling model of deformation.

## Results

**Figure 1:**
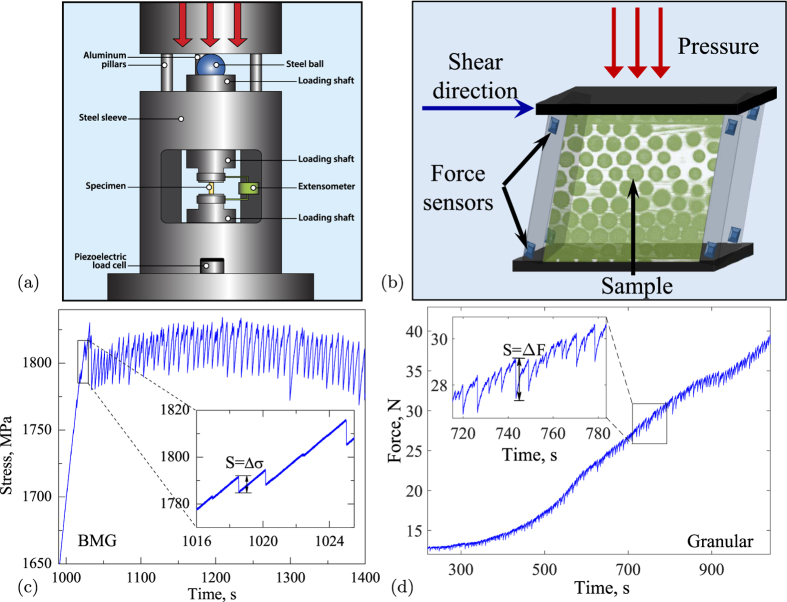
Metallic glass and granular setups and measurements. (**a**) Schematic of the bulk metallic glass measurement setup. Two tungsten carbide platens that are constrained by a steel sleeve compress the metallic glass specimen, see ref. 3 for details. (Drawing is courtesy of Adrienne Beaver, Bucknell University). (**b**) Schematic of the granular shear cell setup with force sensors in the walls. Loads imposed on top exert a constant confining pressure, see ref. 7 for details. (**c**,**d**) Metallic glass and granular data - the main panels show applied stress or force versus time, insets show magnifications of the data.

Due to their very different nature of hard versus soft solids, metallic glasses and granular materials differ greatly in mechanical properties such as modulus, ductility and elastic strain. To nevertheless resolve and compare the fine fluctuations of the applied stress in the two systems, we developed specific experimental protocols for each^19^,^7^. For the metallic glass specimens we applied uniaxial compression tests at constant displacement rate with a nominal strain rate of 10^−4^ *s*^−1^ using a precisely aligned load train with a fast-response load cell and high-rate data acquisition, see Fig. 1(a) and Methods for details. We confirmed that the results are robust with respect to changes in the metallic glass composition and strain rate^6^. During compression the specimen deforms elastically until a shear band or slip event initiates. This causes the displacement rate to temporarily exceed the displacement rate imposed on the specimen, resulting in a stress drop as shown in Fig. 1(c), inset^19^. The size of the stress drop is proportional to the slip size. The granular material is deformed at constant shear rate using a shear cell with built-in pressure sensors, held at constant pressure by a confining top plate, see Fig. 1(b)^ ^7 and Methods. This shear geometry is the natural choice for a hard-sphere granular system that is not held together by attractive interactions. Since for the metallic glass, compression translates into shear along ~45° inclined planes^20^,^21^ this difference in loading conditions does not affect the slip step distributions and dynamics. We apply confining pressures between 4 and 10 kPa that keep the granular material in a jammed, solid state, resulting in particle volume fractions of around 60%. The granular material is sheared at a constant rate 
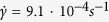
 and force drops are identified around the monotonically increasing average force, as shown in Fig. 1(d), inset. Previous experiments have shown that this strain rate is sufficiently slow to be able to separate individual avalanches and avoid avalanche overlap^7^. These experiments also showed the slip-size power-law distribution remained robust from start-up to steady-state deformation. The number of granular particles is large compared to typical laboratory granular studies, but of course many orders of magnitude smaller than the number of atoms in the metallic glass specimens. The granular linear system size of ~70 particle diameters across can lead to significant truncation of large avalanches and hence to more pronounced finite size effects than for the metallic glass. Both granular and metallic glass systems are strained sufficiently slowly to detect small and large avalanches, necessary to reveal universal behavior in the slip mechanism^22^.

To describe the stress fluctuations in both systems, we use a simple analytic model that predicts the slip statistics and dynamics for elasto-plastic solids^17^,^23^. The model assumes that real solids have elastically coupled weak spots, which are known as shear transformation zones in a metallic glass. Each weak spot slips by a random amount when the local stress exceeds a threshold. Due to long-range elastic interactions in the solid material, a slipping weak spot can trigger other weak spots to slip as well in a slip avalanche, causing the intermittent response that is observed in experiments. For bulk metallic glasses and granular materials, the model assumes that a recently slipped weak spot is slightly weaker than before, due to dilation^19^. As a result, the model predicts a universal power-law scaling for small slip avalanches in a range of sizes that is not affected by finite-size effects of the specimen. The model also predicts the dynamics of the avalanches as jerky velocity-time profiles with a smooth average shape, taking into account material-dependent damping effects, and the setup-dependent machine stiffness. We use the model predictions for these avalanche profiles and their scaling behavior to test universality across frictional dynamics in granular materials versus dynamics with weakening or inertia in BMGs.

For large avalanches, in contrast, the model predicts very different dynamics^14^,^23^. Both frictional weakening (as expected for granular materials) and inertial stress overshoots (as may be present in BMGs) are predicted to lead to almost periodically recurring large avalanches spanning a macroscopic fraction of the system. The individual large avalanches exhibit smooth velocity-time profiles. The model predicts how the average slip avalanche size for the smaller slips grows as a large slip is approached^7^. The model also predicts numerous scaling laws, for example how the statistics changes with applied strain rate and stress, allowing us to extrapolate from one loading condition to another.

**Figure 2:**
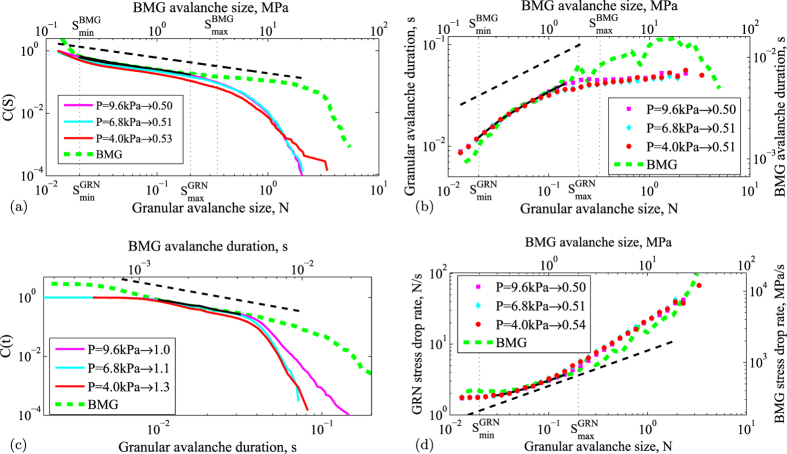
Avalanche sizes, durations and rates. Four scaling parameters are compared for the metallic glass (green dashed line) and the granular material (colored lines with color denoting confining pressure): (**a**) Complementary cumulative distribution *C(S*) of avalanche size, (**b**) Avalanche duration versus avalanche size, (**c**) Complementary cumulative distribution *C(t*) for avalanche duration, and (**d**) Stress drop rate versus avalanche size. In each plot the solid black line shows the portion of the granular data corresponding to the scaling regime (
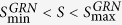
) for data collected at 9.6 kPa. The dashed black line shows the slope expected from the prediction of the mean field model. The legends show the slope value of the granular curves in the scaling regime for pressures 4.0, 6.8 and 9.6 kPa.

The raw stress-time data of the two systems in Fig. 1c,d show pronounced stress fluctuations; rapid stress drops demarcate stress relaxation events, during which the displacement rate temporarily exceeds the displacement rate applied to the specimen. We define the size of these avalanche events *S* from the magnitude of sharp stress drops Δ*σ* and force drops Δ*F*, and the duration *t* as the time passed from the beginning to the ending of a stress drop or force drop in the metallic glass and granular systems, respectively, (see insets of Fig. 1c,d). Due to the very different particle size (angstroms for atoms in the metallic glass versus millimeters for the granular particles), and the different nature of interaction (atomic potential versus frictional contacts), the magnitude and duration of stress fluctuations differ greatly, being several ten megapascals and several milliseconds for the metallic glass, opposed to several hundred pascals and several ten milliseconds for the granular material. Nevertheless, we can collapse the stress drop distributions by simple rescaling that accounts for the different stress magnitudes of the hard metallic and soft granular materials. To show this, we plot selected rescaled distributions of stress drop magnitudes and durations in Fig. 2. The probability of stress drops larger than size *S*, known as the complementary cumulative size distribution, is shown in Fig. 2a. The metallic glass and granular distributions show overlap for avalanche sizes *S* in the range 
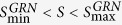
 that are not affected by the sample boundaries. In this small-avalanche regime, both distributions closely follow a power law *C(S*) ~ *S*^−(*τ*−1)^ with exponent *τ* − 1 = 1/2, in agreement with predictions by the mean field model (dashed line). Previous internal imaging of the granulate has shown that this scaling of force drops is indeed directly related to a hierarchical strain distribution inside the granular material, giving direct evidence of the near-critical state of the system^7^. Interestingly, the granular distribution approaches that of the metallic glass and the model predictions *τ* = 3/2 with increasing confining pressure that pushes the granulate deeper into the jammed solid regime (with volume fraction close to 60%). This is because, unlike the metallic glass that is held together by attractive molecular interactions, the granular particles have repulsive interactions and are held together merely by the applied confining pressure. We note that in the large-avalanche regime, the metallic glass and granular distributions show different trends. Here, the granular power-law distributions are truncated due to finite-size effects: they extend over significantly shorter ranges than those of the bulk metallic glass that follows the mean-field prediction up to larger avalanches. We thus identify the small-avalanche regime not affected by finite size effects as the scaling regime of the model, where the overlap of the distributions and model predictions is notably good.

The similar scaling of small avalanches is further confirmed in their duration, as shown in Fig. 2b where we plot the duration as a function of size. For small avalanches with size 
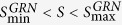
, the agreement between the metallic glass and granular data is very good: both exhibit similar scaling of the size-dependent duration close to 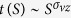 with 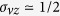 as predicted by the model^17^. Again, this scaling regime extends to larger avalanches for the metallic glass due to its larger system size. For the granular material, the data crosses over to a second nonuniversal scaling regime 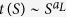 with a much smaller exponent 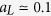. This much shallower growth of the avalanche duration indicates large slip events, common for shear bands or cracks, for which uniform sliding occurs along the entire shear plane. Indeed, it is known that granular materials always have shear bands^24^. Our bulk metallic glass experiments also exhibit shear bands^4^,^25^, but due to the very different boundary conditions the system-specific second scaling regime with small *a*_*L*_ ~ 0.1 is not observed for the metallic glass.

We further explore the correspondence of avalanche durations by plotting their complementary cumulative distributions in Fig. 2c. We again find good agreement in the small-avalanche regime that again improves with increasing confining pressure: the metallic glass and highly confined (*P* = 9.6 kPa) granular system exhibit identical power-law distributions with the predicted slope of −1. Similar to Fig. 2a, the scaling regime extends to larger avalanches for the metallic glass, while for the granular system a second scaling regime emerges that clearly changes with the applied confining pressure, and is thus a non-universal regime that depends on the system details.

A characteristic property of the avalanche dynamics is the rate of stress release, which gives insight into the propagation of individual avalanches. Plotting the rate of stress release as a function of avalanche size, we find very good agreement between the metallic glass and granular data over the entire avalanche regime (Fig. 2d), signifying that the underlying avalanche propagation dynamics for small avalanches may be the same in both systems. Yet, the scaling range of the stress drop sizes that can be compared to mean field theory is limited by finite size effects.

**Figure 3:**
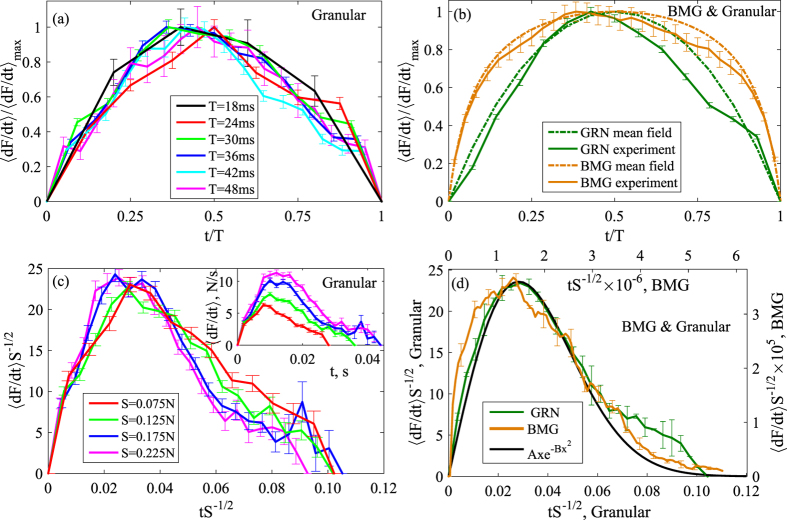
Temporal avalanche profiles in the universal scaling regime. (**a**) Average stress-drop rate normalized by the maximum rate of the granular system at the highest confining pressure. Profiles are averaged over avalanches from small bins of their durations. Error bars are calculated as standard error of the mean. (**b**) Stress-drop rates compared for metallic glass (brown) and granular material (green). Solid lines show averages over avalanches in the scaling regime. Dash-dotted curves show mean-field predictions with *kT* ≈ 9 for the metallic glass data and *kT* ≈ 0 for the granular data. (**c**) Granular stress-drop rates for fixed avalanche sizes *S* in the scaling regime (
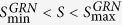
). Inset shows original data, and main panel shows collapsed profiles scaled along both axes by *S*^−1/2^. (**d**) Comparison of the averaged collapsed profiles for granular data (**c**) and metallic glass data^19^. The black curve shows scaling functions predicted by the mean-field model for both granular materials (bottom axis) and metallic glasses (top axis), which perfectly overlap with each other, further corroborating the similarity of the slip avalanche statistics of metallic glasses and granular materials. The granular fitting constants for the scaling function *Ax* exp(−*Bx*^2^) are *A* = 1.46 and *B* = 6.6 × 10^−4^; the metallic glass constants are *A* = 3.98 × 10^11^ and *B* = 2.18 × 10^11^.

The advantage of our systems is that we can use the finely resolved signals to compare the full time evolution of the avalanches. We show the rate of force release as a function of time for avalanches in the scaling regime in Fig. 3. As expected for the scaling regime, we can indeed collapse all granular avalanche profiles with different durations onto a single master curve as shown in Fig. 3a. A similar collapse for the metallic glass avalanches has been shown in ref. 19. This self-similarity lends credence to the idea that in this regime the system is indeed described by robust scaling relations.

We compare the granular data with that of the metallic glass and mean-field predictions in Fig. 3b. While the profiles appear similar, they exhibit characteristic differences. For the metallic glass, the avalanches show a symmetric profile, in good agreement with mean-field predictions, and a flat shape indicating finite machine stiffness. We fitted these profiles using the scaling function^19^,^26^ 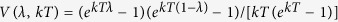 with fitting parameter *kT* ≈ 9, where *T* is the total duration of the avalanches, *k* is the rate associated with machine stiffness and finite size effects, and *λ* = *t/T*. For the granular material, these profiles are less flat, well described by *kT* ≈ 0, close to the simple parabolic form *t/T* · (1 − *t/T*), indicating that no machine-related effects interfere with the avalanche propagation in the granular experiment. The avalanche shape is asymmetric and more tilted to the left than for the metallic glass, suggesting delayed damping effects^27^ similar to earthquakes^28^. Delay effects can originate with time scales inherent to the friction between the granular particles. As the particles are relatively soft, their elastic relaxation time that sets the microscopic delay time for the onset of slip is considerable. A similar explanation has been suggested for earthquakes^28^, and for Barkhausen noise in magnetic materials, where the delay is due to eddy currents in the material^27^,^29^. Recent simulations show similar asymmetric avalanche shapes in interface depinning processes^30^ and in deformed amorphous materials^31^. In contrast, in metallic glasses there is no friction between the atoms inside the alloy, and consequently no significant microscopic delay time to yield a noticeable skewing of the avalanche shapes, for which only a small negligible tilt to the left is detected. The absence of the tilt to the right also means that the inertia does not influence small BMG or granular avalanches. In either case, the asymmetry of the velocity profile does not affect the scaling exponents; they are still given by the mean field model predictions as shown in ref. 32.

The reason why the avalanche shapes are not as flattened for the granular material as they are for the metallic glass is the relatively small machine stiffness. For the metallic glass experiments the machine stiffness was chosen to be large^19^, leading to a broadening of the avalanche shape^19^,^27^. In the granular experiments the walls are of similar stiffness to the granular particles, thus not significantly flattening the temporal profile. By fitting the predicted form to the granular data using *k* = 0 (no influence from the machine stiffness) we find good agreement between model predictions and experiments as shown in Fig. 3b. In summary, our finely resolved measurements of the avalanche profiles reveal the details of machine stiffness and avalanche delay effects due to interparticle friction^27^ within the same scaling and universality class.

We can now test the full universal scaling of the avalanche dynamics. We do this by collapsing profiles as a function of avalanche size, focusing on the small-avalanche regime. Individual profiles are shown in the inset of Fig. 3c. These profiles indeed collapse onto a single master curve when we rescale both axes by *S*^−1/2^ (main panel of Fig. 3c), indicating that the scaling property applies to the full dynamic profiles. The average of these profiles agrees well with that of the metallic glass, and closely matches the prediction by mean-field theory, as shown in Fig. 3d, suggesting that avalanche dynamics do indeed have universal properties. The small difference between metallic glass and granular data for small values of *tS*^−1/2^ < 0.02 is due to differences in particle softness and machine stiffness, similar to Fig. 3b. Consequently, the metallic glass profiles also can be fitted with the mean-field theory using different values of the non-universal parameters *A* and *B*^19^, but the form of the two scaling functions *Ax* exp(−*Bx*^2^) for granular and metallic glass data can be perfectly overlapped with each other when plotted in their corresponding axes (Fig. 3d). While some small deviations for the granular avalanches occur at large values of *tS*^−1/2^ > 0.06 due to poor statistics, the overlap of granular and metallic glass data, and the mean field prediction is striking. We thus conclude that this small-avalanche regime shows strong similarity not only in the statistics, but also in the full avalanche dynamics.

**Figure 4:**
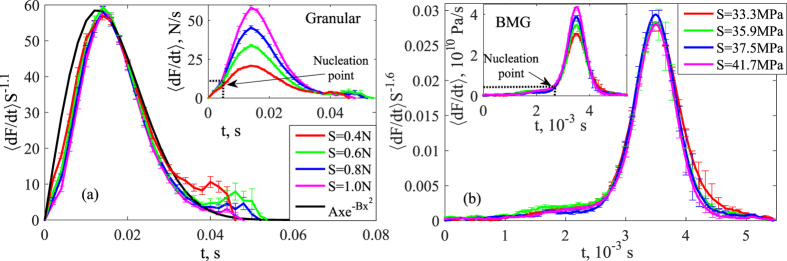
Temporal profiles of large granular avalanches. Stress-drop rate profiles of large avalanches for the granular (**a**) and metallic glass systems (**b**). Profiles averaged by size are shown in the insets, and collapsed data is shown in the main panels. Error bars are calculated as standard error of the mean. (**a**) Large granular avalanches show good collapse when scaling the vertical axis by *S*^−1.1^. Scaling along the horizontal axis is not required. The mean-field scaling function *Ax* exp(−*Bx*^2^) can be fitted with *A* = 7.4 and *B* = 3 × 10^−3^. The nucleation point at *t* ≈ 7 · 10^−3^ *s* and 〈*dF/dt*〉 ≈ 10 *N/s* when the small avalanche turns into a large one is very close to a maximum point of the small avalanche profiles shown in Fig. 3c. (**b**) Large metallic-glass avalanches show collapse when the vertical axis is scaled by *S*^−1.6^. Along the horizontal axis, the profiles have been centered manually at the peak positions. In the large-avalanche regime, the scaling of the avalanches is not universal, as it is affected by the different boundary conditions of the systems, in agreement with mean-field predictions.

In contrast, the second scaling regime for large avalanches 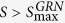 shows non-universal dynamics that depend on the system geometry and boundary conditions, as indicated by Fig. 2a,c and consistent with the model predictions. We can still collapse the large avalanche profiles for the granular system: taking the raw profiles (inset in Fig. 4a) for maximum confining pressure, we achieve good collapse by scaling the vertical axis by *S*^−1.1^, as shown in the main panel of Fig. 4a. Scaling along the horizontal axis is not required due to the almost constant avalanche duration (Fig. 2b). Although the predictions that mean field theory makes for the small avalanches are not expected to necessarily extend all the way to the large avalanches that are affected by system boundaries and loading geometries, the collapsed granular profiles are also in good agreement with the small-avalanche mean-field scaling function *Ax* exp(−*Bx*^2^) indicated by the black line.

Finally, these large-avalanche profiles allow us to elucidate the connection to the small-avalanche regime: initially (*t* < 8 · 10^−3^ *s*), these profiles exhibit a “foot” that corresponds precisely to the profile of the small avalanches shown in the inset of Fig. 3c. When reaching their maximum 〈*dF/dt*〉 ≈ 10 *N/s* at *t* ≈ 7 · 10^−3^ *s*, some of these small avalanches nucleate into larger ones, upon which the avalanche size grows faster, as clearly seen in the profiles in Fig. 4a, inset. This nucleation picture of large avalanches is consistent with the mean field model^14^,^17^,^23^. For the metallic glass, this foot is very long^25^ (see inset of Fig. 4b where profiles are centered at the peak positions). Neglecting the foot, we can achieve a reasonably good collapse along the vertical axis by scaling with *S*^−1.6^, which is quite far from the mean-field scaling *S*^−0.5^ for the small avalanches. This is not surprising, since the mean-field theory predicts that the *S*^−0.5^ scaling only applies to the small avalanches but not for the large ones. We associate this difference in the granular and the metallic glass behavior with the difference in boundary effects and loading conditions of the two systems: because the large avalanches feel the system boundaries, and the boundary conditions in both experiments are different, it is expected that the large avalanche profiles in these systems would be different. Note that inertia and/or weakening effects also likely play a role in the formation of the large events in metallic glasses. We hence identify this empirical scaling behavior in the large-avalanche regime as non-universal and system-specific, different from the small avalanche scaling regime that exhibits universal statistics and dynamics.

## Conclusion

We have demonstrated strong similarity of statistics and dynamics of slip avalanches in two disparate systems: metallic glasses and granular systems. Despite their very different particle size and interactions, and the resulting orders of magnitude different stresses, the distributions reveal strikingly similar dynamics and statistics with identical power-law behavior of avalanche sizes and durations. Reaching beyond just avalanche statistics in granular materials, these results for the first time give evidence of universality also in the dynamics (and scaling functions) of avalanches. While our systems are the only ones for which the required time resolution for full temporal tracking of slip avalanches can be currently achieved, we expect many more systems to show similar dynamics.

These results imply that it is possible to predict the deformation dynamics of macroscopic granular materials from experiments on millimeter sized bulk metallic glass samples. This is supported by the fact that the scaling exponents and scaling functions in our experiments agree with predictions of the simple coarse grained mean-field model^23^ that describes the slips as avalanches of slipping weak spots, without regard for material scale or structure. Hence, the model suggests that the results are transferrable to a much larger range of scales, potentially including earthquakes.

These results provide a crucial step forward towards a full universal understanding of the deformation of amorphous materials. By going beyond previous comparisons of slip statistics alone^6^, the current study compares the *dynamics* of slip in terms of both scaling exponents and scaling functions across two systems with completely different scales, structures, and interactions. The good agreement between the systems, and between the experimental data and mean-field predictions resolves an ongoing debate about the slip dynamics and the role of frictional weakening, damping and inertia. Our results suggesting that both avalanche statistics *and* dynamics are universal expand the claim that these systems may be described by a unifying theory of slip-avalanches.

## Methods

For the metallic glass specimens we applied uniaxial compression tests using a precisely aligned load train with a fast-response load cell and high-rate data acquisition, see Fig. 1(a). We used a bulk metallic glass with composition *Zr*_45_*Hf*_12_*Nb*_5_*Cu*_15.4_*Ni*_12.6_*Al*_10_, and specimens 6 mm long along the loading direction with a cross section of 1.5 mm × 2 mm. During compression, the specimen deforms elastically until a shear band or slip event initiates. This causes the displacement rate to temporarily exceed the displacement rate imposed on the specimen, resulting in a stress drop as shown in Fig. 1(c), inset^19^. The size of the stress drop is proportional to the slip size. For the granular system, we used a shear cell with built-in pressure sensors to record the force fluctuations on the tilting walls, as shown in Fig. 1(b)^ ^7. The granular particles, around 3 · 10^5^ polymethyl methacrylate spheres with a diameter of *d* = 1.5 mm and a polydispersity of ~5%, are confined by a top plate, subjected to confining normal pressure between 4 and 10 kPa, resulting in a particle volume fraction of 55–60%. The granular material is sheared at a constant rate 
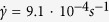
 to a total strain of *γ* = 20%, and force drops are identified around the monotonically increasing average force, as shown in Fig. 1(d), inset. In both systems the stress increases until initiation of another slip event. We measure the stress drops with high temporal resolution to resolve the dynamics of the slip events. This enables us to extract a wide range of predicted scaling exponents and scaling functions that uniquely identify the underlying slip statistics and dynamics.

## Additional Information

**How to cite this article**: Denisov, D. V. *et al*. Universal slip dynamics in metallic glasses and granular matter - linking frictional weakening with inertial effects. *Sci. Rep.* **7**, 43376; doi: 10.1038/srep43376 (2017).

**Publisher's note:** Springer Nature remains neutral with regard to jurisdictional claims in published maps and institutional affiliations.
